# Identification and expression of DoCCaMK during *Sebacina* sp. symbiosis of *Dendrobium officinale*

**DOI:** 10.1038/s41598-020-66616-3

**Published:** 2020-06-16

**Authors:** Yong-Mei Xing, Ming-Ming Zhao, Li-Cheng Guo, Bing Li, Juan Chen, Shun-Xing Guo

**Affiliations:** 1 Key Laboratory of Bioactive Substances and Resource Utilization of Chinese Herbal Medicine, Ministry of Education, Institute of Medicinal Plant Development, Chinese Academy of Medical Sciences & Peking Union Medical College, No. 151, Malianwa North Road, Haidian District, 100193 Beijing, P.R. China; 2 TCM School of Hainan medical University, No. 3, College Road, Hainan, 571199 Haikou, P.R. China; 3 Chinese academy of agricultural engineering planning & design, NO. 41 Maizidian Street, 100125 Beijing, P.R. China

**Keywords:** Plant biotechnology, Plant symbiosis

## Abstract

*Dendrobium officinale* Kimura et Migo is a famous precious medicinal plant in China. Seed and seedling were cultivated with the mycorrhizal fungus *Sebacina* sp. *CCaMK* was initially cloned from *D. officinale* based on a SSH cDNA library of symbiotically germinated seeds with *Sebacina* sp. Phylogenetic analysis was performed among DoCCaMK and other CCaMKs. The particle bombardment technique was used to visualize DoCCaMK-GFP. qRT-PCR and western blot analysis were conducted to determine the tissue expression patterns of DoCCaMK with (SGS) and without (UGS) *Sebacina* sp. Furthermore, the effect of KN-93 on CCaMK expression was also examined. Using NMT the net Ca^2+^ fluxes and the CCaMK concentration were measured during *D. officinale* seed germination. DoCCaMK had the highest homology with *Lilium longiflorum* CCaMK. The DoCCaMK-GFP protein localized in the nucleus and cell membrane. *CCaMK* expression was significantly upregulated after symbiosis with *Sebacina* sp. KN-93 could be used as an inhibitor of CCaMK to inhibit *D. officinale* seed germination. Ca^2+^ influx and the concentration of the CCaMK in the SGS group was significantly more than that of the UGS group. The characterization of *CCaMK* provides certain genetic evidence for the involvement of this gene during seed germination and mycorrhizal cultivation in *D. officinale*.

## Introduction

Most plants form plant-microbe interactions known as symbioses^1^. For arbuscular mycorrhizal (AM) symbioses, fungi help plants acquire nutrients, especially via phosphorus absorption, and thus play a key role in the improvement of plant growth^2^. For nodulation, plants gain nitrogen from bacterial symbionts in the form of ammonia, which can be easily utilized^3^. On the other hand, the fungal and bacterial partners gain carbon nutrients from plants^2^. Therefore, plants benefit from both mycorrhization and nodulation.

In the most common type of mycorrhization of the endo-mycorrhiza, symbiotic fungi penetrate the plant cell^2^. Orchid mycorrhization (OM) and AM are both forms of endo-mycorrhization, thus, to some extent, they share certain common structural characteristics. For OM, symbiotic fungal mycelia penetrate and colonize the root cortex and subsequently form pelotons, which have been regarded as a typical structure in orchids. Furthermore, previous findings have shown that the mycorrhizal-symbiosis mycoheterotrophic orchids shares at least some common properties with AM-forming plants^4^.

CCaMK, with the full name calcium and calmodulin (CaM)-dependent protein kinase, belongs to the calcium/CaM-dependent protein kinase superfamily^5^. It is a serine/threonine (Ser/Thr) protein kinase that is composed of a kinase domain, a calmodulin (CaM)-binding domain and an EF-hand motif-containing neural visinin-like Ca^2+^-binding domain. The activity of the protein is subject to dual regulation by Ca^2+^ and Ca^2+^/CaM^6^. CCaMK is unique among Ca^2+^-regulated proteins and is able to bind both free Ca^2+^ and Ca^2+^ in complex with CaM. It binds to Ca^2+^ either directly through the EF-hand domain or indirectly via the CaM-binding domain^7^. In recent years, Non-invasive micro-test technique (NMT) is a relatively novel technique to be widely used to record transmembrane ion influx and efflux such as Ca^2+^ directly in a non-contact way by detecting the diffusion potentials outside of the cell membrane in plant physiological research^8^. CCaMKs are plant specific and are distinctly different from calcium-dependent protein kinases (CDPKs) and other serine/threonine kinases in plants, but they are very highly similar to mammalian CaM-dependent protein kinase II (CaMKII)^5^. As an antagonizing protein kinase compound, KN-93 possesses great specificity for the CaMKII class of eukaryotic kinases^9^ and can also moderately inhibit zoospore release, encystment and cyst germination^10^.

*Dendrobium* is one of the largest genera of Orchidaceae. Under natural conditions, seed germination and seedling development of *Dendrobium* require compatible endophytic mycorrhizal fungi to supply many kinds of nutrients in natural conditions^11^. Similar to other precious herbs^12^, Chinese medicinal plant, *D. officinale* has ornamental value and a broad range of therapeutic effects, such as immunomodulation and hepatoprotective activities^13,14^. It is also commonly used as a traditional valuable tonic hygienic food in China^15^. However, due to phytopathogens, pests, commercial overexploitation and anthropogenic interference in natural habitats, this medicinal orchid herb is increasingly endangered^16^. Seed germination is a determining factor in the propagation of pant species^17^. Currently, symbiotic germination and asymbiotic germination are two effective methods for orchid propagation. The former refers to seeds inoculated with mycorrhizal fungi in a relatively barren medium, while the latter refers to seeds sown on sugar-rich medium without fungi. However, symbiotic germination was superior to asymbiotic germination, according to previously reported experiments^18^. Thus, it is imperative to carry out seed and seedling germination and cultivation using techniques that engender symbioses between the host plant and mycorrhizal fungi. Seed germination and seedling propagation in *D. officinale* depend on mycorrhizal fungi, such as Sebacinaceae, Tulasnellaceae and Ceratobasidiaceae, for acquisition of carbohydrates and other nutrients. In recent years, due to symbiosis between mycorrhizal fungi and *D. officinale* seeds or seedlings, increasing attention has been paid to morphological features, biochemical reactions, secondary metabolites and so on^19^, yet the molecular mechanism of *D. officinale* seed germination and seedling growth promotion induced by mycorrhizal fungi is still unclear.

In a previous study, a suppression subtractive hybridization (SSH) cDNA library of symbiotically germinated and ungerminated *D. officinale* seeds was constructed, and as one of the differentially expressed genes, CCaMK was selected as one OM symbiosis-associated candidate gene.

Particle bombardment is a relatively easy and powerful method for the transient expression of genes in plant cells, and transient gene expression has been extensively used in the subcellular localization of fluorescent proteins^20^. Therefore, in this study, the characteristics, subcellular and histological localization, and expression levels of DoCCaMK during *Sebacina* sp. interaction with *D. officinale* seed germination and seedling growth were investigated. Furthermore, a DoCCaMK-GFP fusion protein was also detected using western blot analysis. Whether KN-93 has an antagonistic effect on DoCCaMK during *D. officinale* seed germination and seedling propagation with *Sebacina* sp. was also investigated. In addition, real-time measurement of Ca^2+^ fluxes during *D. officinale* seed germination was detected using NMT technique and CCaMK activity was also investigated. This study will reveal the characteristics of CCaMK and serve as the basis for uncovering the mechanism of CCaMK in OM symbiosis.

## Results

### Homology and phylogenetic analysis of DoCCaMK

According to the SSH cDNA library of symbiotically germinated *D. officinale* seed EST sequences, *DoCCaMK* was found to have high homology (67%) with the *CCaMK* of *Maianthemum racemosum* (MrCCaMK). The full-length cDNA of DoCCaMK (Do807) was 2071 bp long. As deduced by Compute pI/MW, DoCCaMK has 514 amino acids, its isoelectric point is 5.92, and its molecular weight is 57.51 kDa. The comparison results from BLASTX showed that DoCCaMK had the highest homology (81%) with *Lilium longiflorum* LlCCaMK (Q43531). It also had a high homology (78%) with *Brachypodium distachyon* BdCCaMK (XP_003566106) and *Triticum aestivum* TaCCaMK (ADK22086). The identification of conserved domains in the CCaMK protein predicted by InterProScan analyses showed that DoCCaMK possessed serine/threonine/dual-specificity protein kinase catalytic domains, a tyrosine-protein kinase catalytic domain and an EF-hand locus (Table 1).

**Table 1:** Conserved domains and motifs of the deduced DoCCaMK.

Domains and motifs	DoCCaMK
Serine/threonine-protein kinase active-site signature	157-169
Protein kinase ATP-binding region signature	19-44
EF-hand calcium-binding domain	403-415,439–451,481–493
Protein kinase domain	13–296

### Phylogenetic analysis

**Figure 1:**
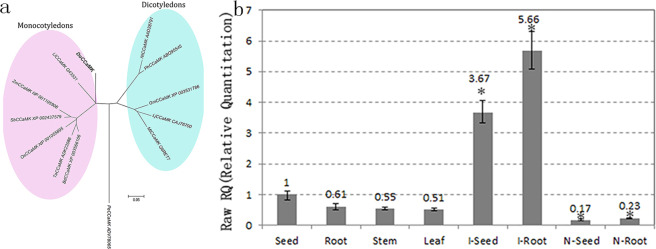
Phylogenetic tree and expression patterns of CCaMK in *D. officinale* by qRT-PCR analysis. In (**a**) Gm: *Glycine max*, Os: *Oryza sativa*, Bh: *Brachypodium distachyon*, LI: *Lilium longiforum*, Sb: *Sorghum bicolor*, Ta: *T. aestivum*, Zm: *Zea mays*, Lj: *Lotus japonicas*, Mt: *Medicago truncatula*, Pe: *Pellia epiphylla*. In (**b**) seed, root, stem and leaf represent different tissues of *D. officinale* without the mycorrhizal fungus, while I-seed and I-root indicate seed and root tissues infected with *Sebacina* sp. N-seed and N-root represent seed and root tissues infected with *Sebacina* sp. after KN-93 treatment. *P < 0.05 (compared to the UGS group).

The phylogenetic analysis was performed using the Neighbour-joining method. The result showed that CCaMKs from monocotyledons and dicotyledons gathered into two separate groups. DoCCaMK and LlCCaMK both belonged to the monocotyledon group and gathered into one clade, which indicated a close genetic relationship between them (Fig. 1a).

### DoCCaMK expression profile analysis

qRT-PCR analysis of the *DoCCaMK* gene at the transcriptional level revealed that in the *D. officinale* tissues without fungus group, *CCaMK* was constitutively expressed and that in the UGS group, it was downregulated in the root, stem and leaf tissues compared to that in the seed (Fig. 1b). *CCaMK* expression was significantly and inducibly upregulated 3.67- and 5.66-fold, respectively, in the seed and root by *Sebacina* sp. induction compared to that in the respective tissues in the UGS group. In the SGS group, after KN-93 was added, *CCaMK* expression levels in the root and seed were both significantly decreased. Furthermore, the expression of *CCaMK* in the KN-93 group was even significantly lower than that in the UGS group (Fig. 1b). The results indicated that *CCaMK* gene expression was induced by the mycorrhizal fungus, which plays important roles in the orchid mycorrhizal system.

### Subcellular localization of the DoCCaMK-GFP fusion protein

**Figure 2:**
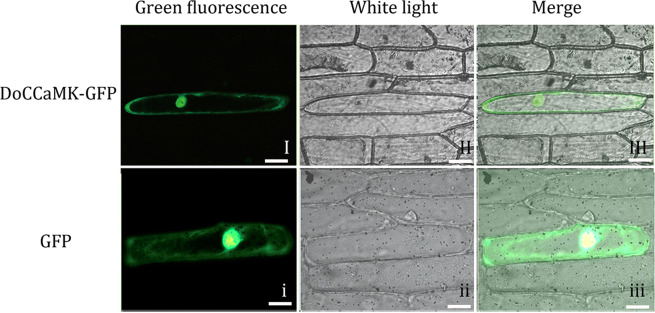
Subcellular localization of the DoCCaMK-GFP fusion protein. Photographs were taken in dark field for green fluorescence (I, i), in bright field for the morphology of the cell (II, ii) and in combination (III, iii). Scale bar: 200 µm.

Using the particle bombardment technique, a transient expression vector was transformed into onion epidermal cells. Then, the localization and distribution of DoCCaMK was observed. The DoCCaMK-GFP fusion protein was observed to be located in the onion epidermal cell nucleus and cell membrane (Fig. 2I) under a fluorescence microscope, while GFP was distributed throughout the onion cell in the control group (Fig. 2i). DoCCaMK-GFP and GFP were illustrated under bright field (Fig. 2II, Fig. 2ii). The DoCCaMK-GFP fusion protein and the GFP protein under white light and merge conditions were shown in Fig. 2III, Fig. 2iii. The results showed that DoCCaMK was localized in the cell nucleus and cell membrane.

### DoCCaMK expression and western blot analysis

**Figure 3:**
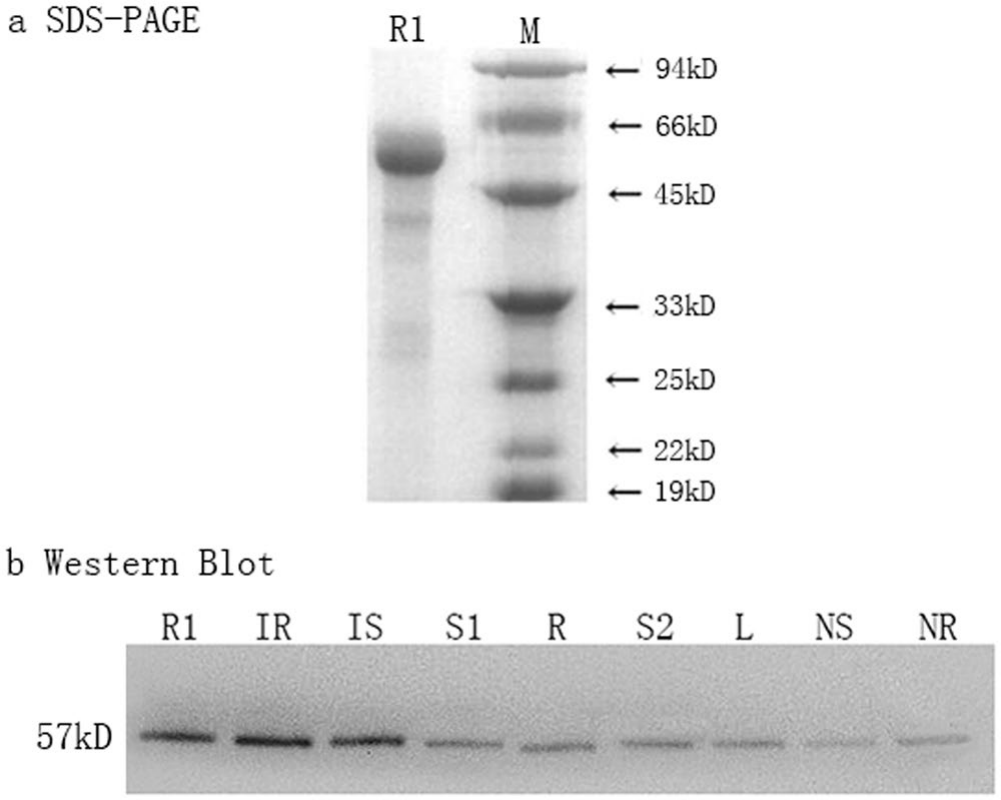
Expression of DoCCaMK in different tissues of *D. officinale* by western blot analysis. In (**a**), M: Low molecular protein marker; in (**a,b**), R1: recombination protein. In (**b**), IR: *D. officinale* root symbiosis with *Sebacina* sp.; IS: *D. officinale* seeds in the SGS group; R: root; S1: seed; S2: stem; L: leaf; NS: KN-93 treatment seeds; NR: KN-93 treatment roots.

SDS-PAGE electrophoresis showed that the molecular weight of the recombinant DoCCaMK protein *in vitro* was 57 kDa, which was in accord with what Compute pI/MW predicted (Fig. 3a). Western blot analysis showed that the expression of CCaMK was different in different tissues of *D. officinale* and that the polyclonal antibody could specifically bind to CCaMK in symbiotically germinated seeds and roots induced by *Sebacina* sp., while it only slightly bound to CCaMK in the seed, stem and root tissues of the UGS group. The molecular weight of CCaMK in the symbiotically germinated seeds and roots was also approximately 57 kDa, which was in accordance with the recombinant DoCCaMK protein *in vitro*. Furthermore, the expression of CCaMK in the seed induced by *Sebacina* sp. was slightly less than that in the root induced by the same fungus (Fig. 3b).

### Effect of KN-93 on *D. officinale* seed germination

**Figure 4:**
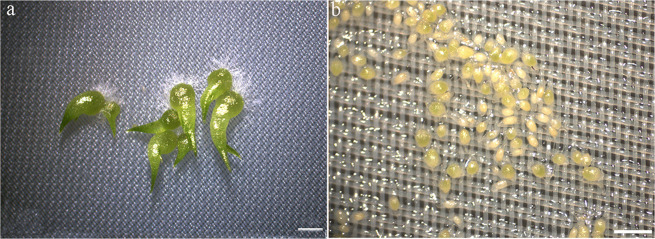
Effect of KN-93 on *D. officinale* seed germination after cultivation for 35 d. (**a**) CK, green protocorms with long shoot tips and rhizoids at 35 d after inoculation (stage 5). Scale bar: 1000 µm. (**b**) In the experimental group, most seeds swelled and geminated to stage 2, but some became only slightly swollen and remained at stage 1. Scale bar: 500 µm.

The dust-like seeds of *D. officinale* depend on mycorrhizal fungi for germination in the similar way as *Gastrodia elata*. At the beginning of germination, Orchids always form protocroms^21^. The developmental stages of seed germination were previously divided into five stages, according to Stewart *et al*.^22^. For example, at the germination stage, the embryo swells, enlarges and emerges from the coat, which is then named stage 2. When the embryo differentiates into a protomeristem, it is called the protocorm stage or stage 3, while at the time of stage 4, the first leaf emerges and the developmental process enters the seedling stage. In the present study, after cultivation for 35 d, *D. officinale* seeds in the SGS group germinated to stage 5, in which shoot tips began extending increasingly longer, and large amounts of rhizoids formed (Fig. 4a). However, in the control group, most of the seeds germinated to stage 2, in which most of the embryos became swollen, turned green and broke through the seed coats, but some only became slightly swollen, with their seed coats remaining intact and staying in stage 1 (Fig. 4b).

### Real-time measurement of Ca^2+^ fluxes during *D. officinale* seed germination

**Figure 5:**
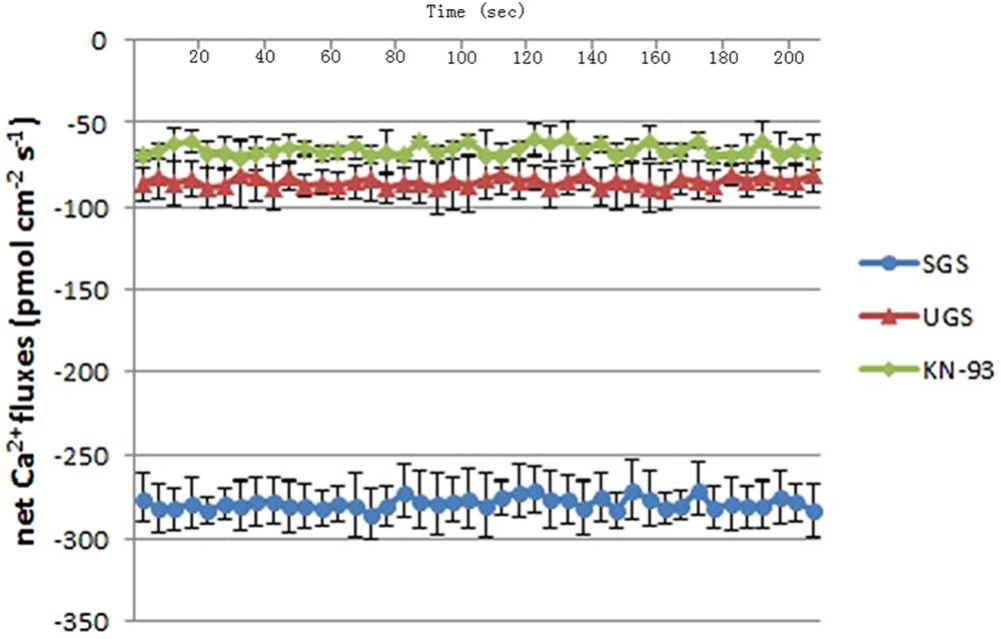
Real-time measurement of Ca^2+^ fluxes during *D. officinale* seed germination Ca^2+^ fluxes (pmol·cm^−2^·s^−1^) showed that the negative values standed for influx of Ca^2+^ and the positive values represented for efflux of Ca^2+^. The values of net Ca^2+^ flux were means ± SD (n = 6). After inoculation for 35d, Ca^2+^ influx in the seed continued for a period of time in different groups during *D. officinale* seed germination and the net Ca^2+^ uptake in the SGS group increased significantly than that of the UGS and KN-93-treatment group.

As shown in Fig. 5, after seed germination for 35d, in the SGS, UGS and KN-93 complement groups, the result showed that the uptake of Ca^2+^ in the SGS group was evidently increased compared to that of the UGS and KN-93 complement groups. The mean Ca^2+^ influx was lower 229% and 320% in the seed of the UGS group and KN-93 complement group than that of the SGS group.

### CCaMK concentration detection during *D. officinale* seed germination

**Figure 6:**
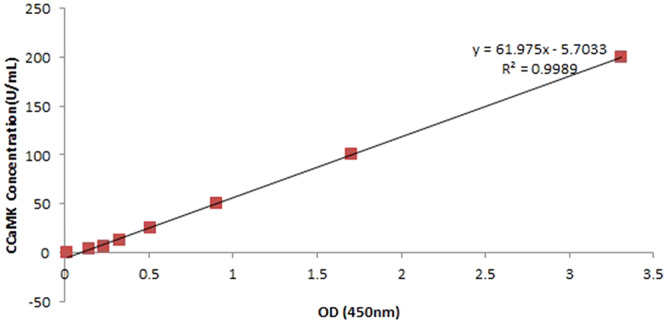
The standard curve equation of CCaMK concentration. The standard curve equation of CCaMK indicated that within a CCaMK concentration range from 3.125 to 200 U/ml, the corresponding absorbance values showed a reliable linear relationship.

The standard curve and regression equation were established (y = 61.975×−5.7033, R^2^ = 0.9989) in Fig. 6. The concentration of CCaMK in the SGS group was 14.17 U/mL, significantly higher than that of the control (UGS) group (*P* < 0.05) with 2.45 folds change. The CCaMK activity of the UGS group was 1.61 times that of the KN-93 group (Table 2).

**Table 2:** The CCaMK activity in the UGS, SGS and KN-93 groups.

Group (n = 3)	CCaMK activity (U/mL)
UGS	5.99 ± 0.52
SGS	14.17 ± 0.16*
KN-93	3.74 ± 0.10*

All the data were analyzed with one-way ANOVA. Significant differences were determined using Student-Newman-Keuls method. The values were presented as the means ± SD with 3 replicates of each group, from at least three independent experiments, *P < 0.05 (compared to the control group).

## Discussion

As an important signalling molecule in eukaryote cells, CCaMK plays a critical role in many physiological processes. In plants, it participates in development and hormone-related reactions, mainly involving light response regulation, stress and symbiosis. For example, during *T. aestivum* seed germination and seedling development, *CCaMK* overexpression decreases the sensitivity of the host plant to abscisic acid (ABA)^13^. In recent years, *CCaMK* has been proven to exert vital regulatory effects in the signal transduction of the symbiosis interaction between rhizobia or arbuscular mycorrhizal fungi and many plants^23^, while *Arabidopsis* has been reported to be the only non-mycorrhizal plant species, lacking the *CCaMK* gene^24^. The phylogenetic analysis in the present study showed that DoCCaMK had the highest homology with LlCCaMK, followed by that with BdCCaMK and TaCCaMK, with a homology of 78%. Therefore, plant CCaMKs are structurally conserved at both the protein and gene levels^5^.

It was found that the induction of the host genes essential for infection of AM fungi required nuclear localization of *CCaMK*^23^. A previous study also reported that ZmCCaMK is located in the nucleus^25^. Transient transformation of the epidermal cell layer of the onion bulb with the particle bombardment method and subcellular localization analysis of CCaMK fused with green fluorescent protein in the present study showed that DoCCaMK-GFP of *D. officinale* was located in the nucleus and the membrane, which was slightly different from what had been predicted before, with localization only in the nucleus^25^. As Ca^2+^ in the nucleus requires interactions between K^+^ channels, Ca^2+^ transporters and Ca^2+^ channels at the membrane of the nucleus^26^, CCaMK localizing in the cell membrane and nucleus of *D. officinale* may facilitate coordinated activities among ions.

It is well known that specific genes are involved in a so-called common symbiotic pathway (CSP)^3^. As CCaMK is considered to be a master decoder, a regulatory kinase and a key component of the CSP, it plays an essential role in deciphering the nuclear Ca^2+^ oscillations to control the proper transcriptional response^2^. Calcium oscillation requires CCaMK, and the activation of CCaMK is sufficient not only for AM and rhizobium-legume symbioses but also for actinomycetes to induce symbiotic processes^27^. The occurrence of calcium spiking, which caused the intracellular calcium ion concentration oscillates will response to nodulation or mycorrhization factors produced by the microbial symbionts^28^. In the present study, real-time Ca^2+^ fluxes measurement during *D. officinale* seed germination using NMT showed that Ca^2+^ influx in the SGS group was significantly more than that of the UGS group (Fig. 5). The Ca^2+^ uptake in the SGS group caused intracellular Ca^2+^ concentration to be increased and this may facilitate Ca^2+^ spiking formation and interaction with CCaMK. The more Ca^2+^ uptake from the environment, the more CCaMK will probably interact with this ion. In addition, the concentration of the CCaMK in the SGS group was much higher than that of the UGS group (Table 2), which indicated that the mycorrizal fungus *Sebacina* sp. could increase the CCaMK activity of the host plant.

CCaMK is highly expressed in tissues except for in roots, indicating its other roles in addition to the regulation of symbiosis^29^. Partly consistent with this observation, in the present study, the expression of CCaMK presented tissue specificity, and it was expressed relatively highly in the seed compared to that in the root, stem and leaf in asymbiotic group (Fig. 1b). CCaMK expression was low in the seed, root, and stem of *D. officinale* without interaction with a mycorrhizal fungus; however, it was upregulated 3.67 and 5.66 times, respectively, in the seed and root induced by *Sebacina* sp. compared to that in those tissues in the UGS group. In the SGS group, the expression level of CCaMK, especially in the root of *D. officinale*, was induced much higher than in other tissues, which indicated that *Sebacina* sp. directly and intensely contacted *D. officinale* roots and interacted with them in the co-culture system between *D. officinale* seedlings and the fungus; the result will be confirmed in the undergoing work. Additionally, at the protein level, DoCCaMK was only weakly expressed in the stem, seed and root, but it was highly expressed in the seed and root of the SGS group (Fig. 3b). Furthermore, DoCCaMK was expressed a bit more highly in the root relative to that in the seed induced by *Sebacina* sp., indicating that DoCCaMK probably affected symbiotic interaction with the root of the host plant more remarkably than that in the seed germination. The results indicated that DoCCaMK was most likely induced by the mycorrhizal fungus and participated in OM symbiosis.

In symbiotic seed germination, mutualistic fungi colonize orchid seeds and provide necessary nutrients for protocorm formation^30^. As a Ser/Thr protein kinase, CaMKII is well characterized and widely distributed and has been used as a model for CCaMK due to the significant structural and sequence similarities between the two proteins. For example, the CaM-binding domain of CCaMK shows high sequence identity (79%) to the α subunit of CaMKII, although there are also a few differences in the mechanism of action between them. Similar to CCaMK, autophosphorylation is also key to the regulation of CaMKII, and they both interpret a calcium spiking signal in a similar manner^2^. KN-93 is a synthesized inhibitor of CaMKII^31^. In our present study, we found that KN-93 could also inhibit *D. officinale* seed germination. During the same cultivation period as that for the control group without KN-93, we found that KN-93 could prevent seed germination to stage 5, halting development at stage 2 or even stage 1 (Fig. 4b). qRT-PCR and western blot analyses showed that in the KN-93 complement group, compared to that in the control group, CCaMK was downregulated not only at the mRNA level but also at the protein level in the SGS group (Figs. 1b and 3b). Therefore, KN-93 could inhibit CCaMK during *D. officinale* seed germination induced by *Sebacina* sp. In addition, in comparison to that of the SGS group, KN-93 could also decrease Ca^2+^ influx and reduce CCaMK activity during *D. officinale* seed germination. The results of the present study demonstrated that CCaMK participates in *D. officinale* seed germination. However, the interaction of other molecules with CCaMK in symbiotic germination of orchid seeds and the symbiotic mechanism between the orchid root and mycorrhizal fungus deserves further research.

## Conclusions

CCaMK has been reported to play important roles in AM and nodulation symbioses, but little is known about its role in the OM. In this study, DoCCaMK-GFP fusion protein is found to locate in the cell nucleus and cell membrane. After *Sebacina* sp. colonization, CCaMK was highly expressed not only in the root, but also in the seed of *D. officinale*. In addition, during seed germination, the Ca^2+^ influx and the concentration of CCaMK was more in the SGS group than that of the UGS group. KN-93 can prohibit seed germination and inhibit CCaMK expression, furthermore, it can also reduce CCaMK activity and the Ca^2+^ influx during seed germination. This investigation of the characteristics and the expression patterns of CCaMK in *D. officinale* seed germination and seedling growth will provide key insight into the *D. officinale*-mycorrhizal fungal interaction in the future.

## Methods

### Plant materials, fungal strains and symbiotic germination

Capsules of *D. officinale* were collected from the Menghai experimental base of the Yunnan branch, Institute of Medicinal Plant Development, Chinese Academy of Medical Sciences & Peking Union Medical College Xishuangbanna, Yunnan, China. According to a previous study^19^, seeds in an *in situ* seed baiting technique experiment were divided into two groups. One group of seeds was cultured with *Sebacina* sp., which was isolated from collected germinated seeds according to a previous report^18^. The seeds and *Sebacina* sp. were cultivated in OMA medium (g L^−1^; oatmeal infusion-30 g and agar-15 g) and were defined as SGS. The other group of seeds that was cultured in OMA medium without any fungi was defined as UGS. The seeds were sown and cultured for 5 w in 20 cm diameter pots containing growth medium (bark:pebble:coarse humus = 3:1:1), as previously described^19^. Then, the cultivated samples in the SGS and UGS groups were all collected and immediately frozen in liquid nitrogen prior to RNA extraction.

### Co-culture system between *D. officinale* seedlings and *Sebacina sp*

*Sebacina* sp. was cultivated in sawdust-based medium containing corn (166.0 g L^−1^), sawdust (668.0 g L^−1^), wheat bran (166.0 g L^−1^), KH_2_PO_4_ (2.0 g L^−1^), MgSO_4_·7 H_2_O (0.1 g L^−1^) and sucrose (10.0 g L^−1^). The substrate was then divided into 100 g aliquots in plastic bottles and autoclaved at 122 °C for 120 min^32^. In the SGS group, after *Sebacina* sp. was cultured for 1 month, 3 g of the fungal cultures was then transferred and inoculated next to the roots of three *D. officinale* seedlings in each flowerpot, which were 11 cm in diameter. The components of the medium in the flowerpot were sterile bark, small pebbles and rough humus, with a ratio of 3:1:1. In the UGS group, 3 g of the sawdust-based medium without fungus was added next to *D. officinale*. The experiment was repeated three times, with 30 replicates in each group. After cultivation for 60 d in a greenhouse at 25 °C, the root, stem and leaf in the SGS and UGS groups were collected and frozen in liquid nitrogen prior to RNA extraction and qRT-PCR analysis.

### Total RNA extraction and complementary DNA synthesis

The total RNA of each frozen sample was extracted using an EASYspin Plus Kit (Aidlab, Beijing, China) according to the manufacturer’s protocol.

According to the Clontech SMARTer PCR cDNA Synthesis Kit, the samples in the SGS and UGS groups were used as the template, and ds cDNA was synthesized. Based on the EST sequence of the SSH cDNA library, the primers were designed for cDNA fragment amplification (Table 3). PCRs were conducted, and the final amplified PCR products were purified using a QIAquick PCR purification Kit (Qiagen, Hiden, Germany) and directly cloned into the pGEM-T Easy vector (Promega, Madison, WI, USA), which was then transformed into competent *Escherichia coli* DH5α cells (Trans, Beijing, China). Transformed cells were then screened by the blue/white colony method^19^.

**Table 3:** Gene-specific primers used in different reactions.

Primers	Forward	Reverse
cDNA synthesis of CCaMK	GACCATCTAGTTTCAAAACCC	CATTCATTACAAAGCGTGC
Cloning of DoCCaMK	GSP1: CATCTTCCACAATCCGTCTCATCACC GSP2: CTAAGCCGCAAGCTATCTGGCGCAC	GSP3: GGGCAGAAACCTCCAACATTGAAGCA GSP4: GATTCTGGTGATGAGACGGATTGTGG
qRT-PCR	CACTCAAAAGGTTAGGGTTCAT	TCTCATTAGTCAGCAAAGCATC
Construction of CCaMK-GFP fusion expression vector	GTCGACATGTCGAGCCTGGAGAATAGAAAG	GGATCCGTTAGGGCGCAGTGTGGAGA
CCaMK-TE	GGATCCATGTCGAGCCTGGAGAATAGAAAG	GTCGACCTAGTTAGGGCGCAGTGTGGAG

### Cloning and characterization of the full-length DoCCaMK gene from *D. officinale*

Isolation of the 3′/5′-end of *CCaMK* was performed using a SMART RACE cDNA Amplification Kit (Clontech, Japan) according to the manufacturer’s protocol. Based on the sequences of the SSH cDNA Library, four gene-specific primers (GSPs) were designed (Table 3).

The amplified products were purified, cloned into the pGEM-T Easy vector (Promega, Madison, WI, USA) and transformed into DH5α cells. Positive recombinant plasmids were screened and sequenced by Beijing Genewiz, Inc. After clustering and splicing with the core sequence, the ORFs of each sequence were obtained using BLASTX and ORF finder. Primers targeting the ORF determined in RACE screens were used to sequence and confirm the first reconstituted RACE sequences^19^. The nucleotide sequences of *CCaMK* reported in the present study are available in the GenBank database under accession number MG545931.

### Effect of KN-93 on *D. officinale* seed germination and seedling growth

The seeds and roots of *D. officinale* cultured with *Sebacina* sp. as mentioned above were designated as the control group samples, while the samples of the group that received KN-93 (Sigma-Aldrich Co., St. Louis, MO, USA) treatment at a final concentration of 0.1 μmol/L in OMA medium were referred to as the experimental group samples. In addition, the seeds in the UGS group in OMA medium standed for the negative control group. After inoculation for 35 d, the seed germination in these three groups was subjected to morphological examination using a stereoscopic microscope (Leica DM2500). Additionally, the roots and the seeds in each group were collected for qRT-PCR and western blot analysis. The experiment was repeated three times, with 30 replicates in each group.

### qRT-PCR analysis of DoCCaMK expression

According to the cDNA sequences of *CCaMK* in *D. officinale*, the primers were designed for qRT-PCR using Primer Premier 5.0 (Table 3). To analyse gene expression characteristics, different tissues of *D. officinale* were collected as follows: germinated seeds in the SGS group with and without KN-93 treatment, ungerminated seeds in the UGS group after cultivation in OMA medium for 35 d, and roots of seedlings with and without KN-93 treatment after cultivation with *Sebacina* sp. for 60 d. The roots, stems and leaves of the seedlings without fungus were also collected. qRT-PCR was conducted using a real-time SYBR Green kit (Takara, Dalian, China) and an ABI 7500 Real-Time PCR System (Applied Biosystems, Foster City, CA, USA). *GAPDH* was used as a reference control. The reaction was performed using the following conditions: denaturation at 95 °C for 30 s, followed by 40 cycles of amplification (95 °C for 5 s, 60 °C for 40 s). The gene expression ratio was evaluated by the comparative 2^−ΔΔCt^ method of relative gene expression quantification^19^.

### Phylogenetic analysis

To understand the phylogeny of DoCCaMK, the CCaMK sequences of several plants or crops, such as *Glycine max, Oryza sativa*, *Lotus japonicas*, *Medicago truncatula* and so on, were subjected to phylogenetic analysis. A phylogenetic tree was constructed using MEGA software version 6.0, using *Pellia epiphylla* CCaMK as an outgroup.

### Construction of a plant expression vector and transient expression of a DoCCaMK-GFP fusion protein

Using a 163-hGFP plant expression vector containing 239 amino acids of GFP, an enhanced CCaMK-GFP fusion expression vector was constructed. Plasmid vector DNA (3 µg) was added to 6 µl of a suspension of gold nanoparticles (50 mg mL^−1^) with a diameter of 1.0 µm and mixed evenly. Next, 0.1 M spermidine (4 μL) and 6 μL of CaCl_2_ (12.5 M) were also added and blended thoroughly for 3 min, and then, the mixture was incubated in an ice bath for 15 min. After centrifugation at 12,000 rpm for 10 s, the liquid supernatant was discarded, and ethanol was added and vortexed. Finally, the gold nanoparticle sediment was collected, and ethanol was added to suspend the precipitation for further studies.

Onion bulb scale leaves were cut into strips of approximately 2 cm × 2 cm and were cultured in the centre of MS medium. The fusion expression vector containing the target gene and GFP in the control group was bombarded by the particle bombardment transforming method (1100 psi) to transform the onion epidermis^20^. After transformation, the onion epidermis was cultured in darkness for 24 h and photographed using a Zeiss laser scanning confocal microscope (LSM 510 System, Zeiss, Germany).

### Recombinant protein expression and purification and western blot analysis

Using the pET-28a plant expression vector, the CCaMK-28a eukaryotic expression vector was constructed, and the specific primer sequences (CCaMK-TE) are listed in Table 3. CCaMK-TE-Pet-28a was expressed in *E. coli* BL21 (DE3) competent cells that were grown in 500 mL of LB broth containing 50 μg/mL kanamycin and shaking at 180 rpm at 37 °C until the OD 600 reached 0.8. Next, sterilized IPTG was added at a final concentration of 0.4 mM, followed by shaking at 250 rpm at 37 °C for 4 h. The cultures were then harvested by centrifugation at 10,000 × g (high-speed tabletop centrifuge, Sorvall ST8, Thermo Fisher Scientific Corporation, MA, USA) for 10 min at 4 °C. The pellets were re-suspended in 30 mL of lysis buffer containing 20 mM HEPES, 1 mM NaCl, 2 mM BME, 0.3 mg mL^−1^ lysozyme, and 5 mM imidazole (pH 8); incubated on ice for 30 min; frozen at −80 °C for 10 min; and thawed at 4 °C. After 3 repetitions, the protein was subjected to ultrasonication at 4 °C for 10 min. After centrifugation, the supernatant was collected, purified using a column packed with 1 mL of Ni-NTA resin in a gravity flow chromatography step and equilibrated with lysis buffer. The purified protein was eluted using a gradient of imidazole (10, 20, 80 and 300 mM) in lysis buffer. The purity of the protein samples was estimated by SDS-PAGE^33^.

According to the “one-step plant active protein extraction kit” (Sangon Biotech, Shanghai, China), different tissues of *D. officinale* were collected as mentioned above in the “qRT-PCR analysis of *DoCCaMK* expression” section. After sodium dodecyl sulfate polyacrylamide gel electrophoresis, DoCCaMK was electrotransferred onto a PVDF membrane (Millipore, USA), which was blocked for 4 h with a blocking solution containing 5% non-fat powdered milk. After incubation with a New Zealand rabbit anti-CCaMK primary antibody and an HRP-linked secondary antibody, the PVDF membrane was then developed using an enhanced chemiluminescence detection kit (TransGen Biotech, China) and analysed with a Gel Doc XR + imaging system (Bio-Rad, USA)^33^.

### Real-time Ca^2+^ fluxes measurement of *D. officinale* seed germination using NMT

In this study, cellular Ca^2+^ was measured on the surface of the germinated or ungerminated seed during seed germination on 35d in the SGS, UGS and the samples of the SGS group that received KN-93 as mentioned above using Non-invasive Micro-test Technology System (NMT100 Series, Younger USA LLC, Amherst, MA, USA). Ca^2+^-sensitive microsensor was purchased from NMT Service Center, Xuyue Beijing (Beijing) Sci. & Tech Co. Ltd. The experimental voltage is +600 mV. The primary position (M1) of Ca^2+^-sensitive microsensor was placed 30 μm from the germinated or ungerminated seed surface, and further away position (M2) is 60 μm^34^.

### CCaMK concentration detection during *D. officinale* seed germination

CCaMK standard substance with different concentrations of 200, 100, 50, 25, 12.5, 6.25 and 3.12 (U/ml) was studied. The protocol of the standard curve and the CCaMK concentration detection of the samples were conducted according to the instruction of “Cat No.2Pl-KMLJ91921p plant (SnRKs CCaMK) Camilo ELISA kit”, Camilo biology.

CCaMK activity of *D. officinale* seed during germination was detected and three groups were divided in the experiment. Among them, the samples of the group that received KN-93 treatment at a final concentration of 0.1 μmol/L in OMA medium with *Sebacina* sp. were referred to as the KN-93 complement group. After germination for 35d, 0.2 g of the ungerminated or geminated seeds in triple of each group of the UGS (control), SGS and KN-93 complement groups were respectively collected from the petri dishes, weighed accurately and then grinded with liquid nitrogen. The CCaMK standard substance diluent was regarded as the negative control and the deionized water was treated as the blank control. Subsequently, 1.8 mL PBS buffer solution (0.01 M, pH 7.4) were added and centrifuged at 4 °C, 8000 rpm for 30 min. The supernate of the samples or the CCaMK standard with different concentrations (100 μL) were added to the coated plate and incubated at 37 °C for 90 min. After the plate was washed by TBS washing solution twice and 100 μL of SnRKs CCaMK antibody was added and the plate was incubated at 37 °C for 60 min. Subsequently, the plate was washed for three times and 100 μL of enzyme combo except for the blank control and incubated in the dark for 30 min at 37 °C. The plate was washed for 5 times and 100 μL TMB color solution was added and the reaction lasted for no more than 30 min. At last, 100 μL stop solution was added and the OD (450 nm) was measured in 10 min with EnSpire Multimode Reader (PerkinElmer, America).

## Data analysis

The data were analyzed with one-way ANOVA and the statistical analyse was performed using SPSS 11.0. Data were presented as means ± SD from at least three independent experiments. *P* values < 0.05 were considered significant difference.

## Supplementary information

Supplementary information.
