# Supplementary material for: Identification and expression of DoCCaMK during Sebacina sp. symbiosis of Dendrobium officinale
Source: Sci Rep. 2020 Jun 16;10:9733. doi: 10.1038/s41598-020-66616-3 (PMC7298032; doi:10.1038/s41598-020-66616-3)
Supplement: Supplementary file 1 — Supplementary information. [file 41598_2020_66616_MOESM1_ESM.pdf]

Identification and expression of DoCCaMK during *Sebacina* sp. symbiosis of *Dendrobium officinale*

Yong-Mei Xing[1#], Ming-Ming Zhao[1,2#], Li-Cheng Guo[2,3], Bing Li[1], Juan Chen[1*], Shun-Xing Guo[1*]

[1]Key Laboratory of Bioactive Substances and Resource Utilization of Chinese Herbal Medicine, Ministry of Education, Institute of Medicinal Plant Development, Chinese Academy of Medical Sciences & Peking Union Medical College, No. 151, Malianwa North Road, Haidian District, Beijing, P. R. China 100193; [2]TCM School of Hainan medical University, No. 3, College Road, Hainan, Haikou, P. R. China 571199; [3]Chinese academy of agricultural engineering planning & design, NO. 41 Maizidian Street, Beijing, P. R. China 100125

*Correspondence:

Juan Chen: kibchenjuan@126.com; Shun-Xing Guo: sxguo1986@163.com

[#]Yong-Mei Xing and Ming-Ming Zhao contributed equally to this work.

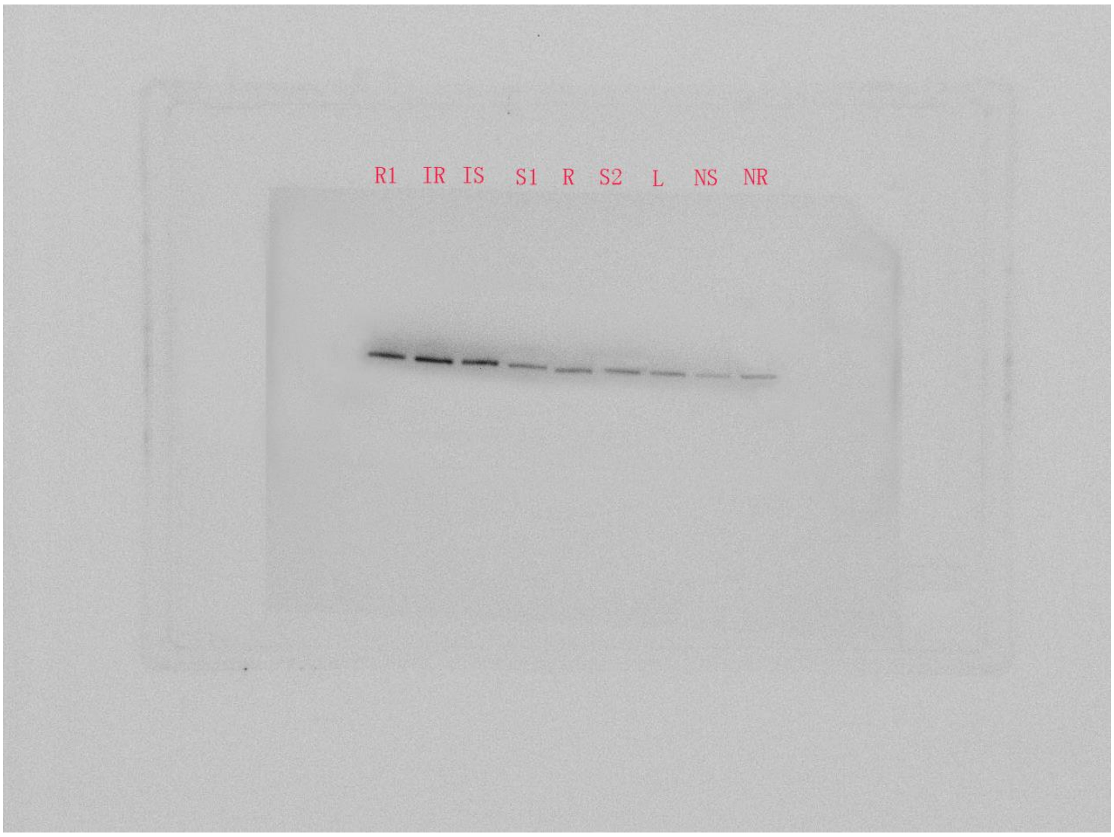

Supplementary Figure 1 Expression of DoCCaMK with Western blot analysis

R1：recombination protein IR: *D. officinale* root symbiosis with *Sebacina* sp.; IS: *D. officinale* seeds in the SGS group; R: root; S1: seed; S2: stem; L: leaf; NS: KN-93 treatment seeds; NR: KN-93 treatment roots.
